# Estimated internal exposure doses due to indoor radiocaesium contamination in residential houses after the Fukushima nuclear accident

**DOI:** 10.1038/s41598-020-74182-x

**Published:** 2020-10-14

**Authors:** Hiroko Yoshida-Ohuchi, Naohide Shinohara

**Affiliations:** 1 Graduate School of Pharmaceutical Sciences, Tohoku University, 6-3 Aramaki-Aoba, Aoba-ku, Sendai, Miyagi 980-8578 Japan; 2 Research Institute of Science for Safety and Sustainability (RISS), National Institute of Advanced Industrial Science and Technology (AIST), 16-1 Onogawa, Tsukuba, Ibaraki 305-8569 Japan

**Keywords:** Environmental sciences, Risk factors

## Abstract

This work first reports the estimation of the internal exposure from ingestion of house dust and inhalation of aerosol, by employing a measured data on ^137^Cs activities, bioaccessibility (solubility to water and 1 M HCl), and particle size distribution. The house dust and aerosol samples were collected during the actual indoor cleaning by vacuuming and dusting, from 65 houses and buildings in proximity to the Fukushima Daiichi nuclear power plant (FDNPP) (1.6–16.1 km from the FDNPP) during a period from April 2016 to January 2019. Committed effective doses for an adult owing to the ingestion of house dust of 20 mg per day, which adheres to one’s hands through the hand-to-mouth, and those owing to inhalation of aerosol during dusting for 1.5 h while wearing a mask, were calculated using DCAL software for each house or building, as 1.13 µSv and 4.55 µSv as maximum doses, respectively (as of March 2011). Both the committed effective doses, owing to ingestion and inhalation, were inversely correlated with the distance from the FDNPP, and positively correlated with the indoor surface contamination.

## Introduction

Following the Fukushima Daiichi nuclear power plant (FDNPP) accident (2011), large amounts of radionuclides were discharged into the environment. During the period in which the radioactive plume passed over the area, the dispersed pollution caused dry deposition in the absence of precipitation. Dry deposition occurred not only outdoors but also indoors owing to the poor air-tightness and high ventilation rate from the leaky wooden Japanese houses, and due to the filtering effect of the building envelop. In certain houses, wild animals such as a wild boar, a civet cat, and a racoon entered and brought soils inside of the houses during the long period of evacuation. Following Decontamination Guidelines^1^ formulated by the Ministry of the Environment of Japan (MOE), decontamination has been carried out outdoors. However, the inside of houses is not subject to decontamination. On March 31, 2017, 6 years after the FDNPP accident, all evacuation orders, except those for Okuma Town, Futaba Town, and the difficult-to-return areas, had been lifted^2^. Successively, evacuation orders were lifted in two regions in Okuma Town on April 10, 2019^3^ and on March 2020^3^ in the towns of Futaba and Okuma that included the difficult-to-return areas around the railway station. This precedes the reopening of the Joban Railway Line all the way through from Tokyo to Miyagi, since the radiation dose in those regions was sufficiently reduced.

Following the revision of the Fukushima Reconstruction and Revitalization Special Measures Law that was approved by the Cabinet on February 10, 2017, the designated reconstruction and rehabilitation base areas, in the difficult-to-return areas, were approved^4^. Further, the evacuation issues are to be lifted in these base areas and the residents will be allowed to return and live within 5 years, taking into account the reduction of the dose. For the residents intending to return to their home and live there, information about the indoor deposition is necessary and important, while assessing the consequences of the nuclear accident in the context of risk assessment, since most of the individuals spend a large portion of their time indoors. Latest statistics show that staying time at home for Japanese individuals is on average 15 h and 15 min, 16 h 49 min, and 17 h and 34 min for weekdays, Saturday, and Sunday, respectively^5^.

The residents are likely to be exposed to indoor contaminants during their daily life, with doses from long-lived contaminants usually being the major long-term hazards.

From July 2013 to January 2015, we investigated the indoor radiocaesium surface contaminants within 95 residential houses in Iitate village, Odaka district, and the towns of Futaba, Okuma, and Tomioka, Fukushima Prefecture, using the smear method. It was revealed that there is a clear distance dependence from the FDNPP, while no distance dependence was seen for outdoor air dose rates caused by wet deposition. For instance, for surface contamination within houses in Iitate village (29–49 km from the FDNPP), 24.8% of samples exceeded the detection limit, which is quite low, whereas in Okuma (< 3.0 km from the FDNPP), 99.7% of samples exceeded the detection limit, and the surface contamination levels exceeded 20 Bq/cm^2^ (as of March 2011)^6^. It was clearly shown that the surface contamination was inversely proportional to the square of the distance between a house and the FDNPP^6^ among residential houses proximate to the FDNPP. Indoor contaminants, which adhere to materials such as house dust and soil, remain indoors and may cause internal exposure by ingestion, while, those that adhere to aerosols are likely to be resuspended by daily behaviors such as vacuuming and dusting, resulting in internal exposure by inhalation as well.

Bioaccessibility should be considered while assessing the internal exposure from the both pathways of ingestion and inhalation. As for the ingestion pathway, bioaccessibility is equal to the absorption fraction from the small intestine to the body fluids, as per the biokinetic model reported by ICRP Publ. 66^7^. As for the inhalation pathway, absorption type (F, M, and S) referring to absorption rates from the respiratory tract into blood in the clearance model reported by ICRP Publ. 72^8^ depends on the bioaccessibility as well. Furthermore, inhalation dose coefficients, which are used to estimate internal exposure, are based on an activity median aerodynamic diameter (AMAD) of 1 µm for the public^9^. Thus, for the next step, it is necessary to reveal solubility (extractability) of house dust and analyzed particle size distribution of aerosol. During a period from April 2016 to January 2019, we sampled house dust by vacuuming from houses and buildings close to the FDNPP, where indoor surface contamination was considered to be rather high, and it revealed extractability to water and 1 M HCl^10^, which is the Japanese official method for soil content measurement for the evaluation of oral exposure^11^. We also sampled the aerosols during dusting, and analyzed ^137^Cs radioactivity concentration in indoor air in each range of the aerosol particle aerodynamic diameters^12^.

Based on these findings, in this study, we report the results of the estimation of internal exposure doses through ingestion of house dust and by inhalation during dusting derived from indoor ^137^Cs contamination, while assuming that the residents return to their home and live in the same way as before the accident. We exhibited a correlation between indoor surface contamination and distance from the FDNPP for examined houses and buildings, and found a correlation between both committed effective doses due to ingestion of house dust and inhalation of aerosol and distance from the FDNPP. Then both the committed effective doses were compared with indoor surface contamination in order to reveal a correlation between them.

## Results

### The correlation between indoor surface contamination and the distance from the FDNPP

**Figure 1:**
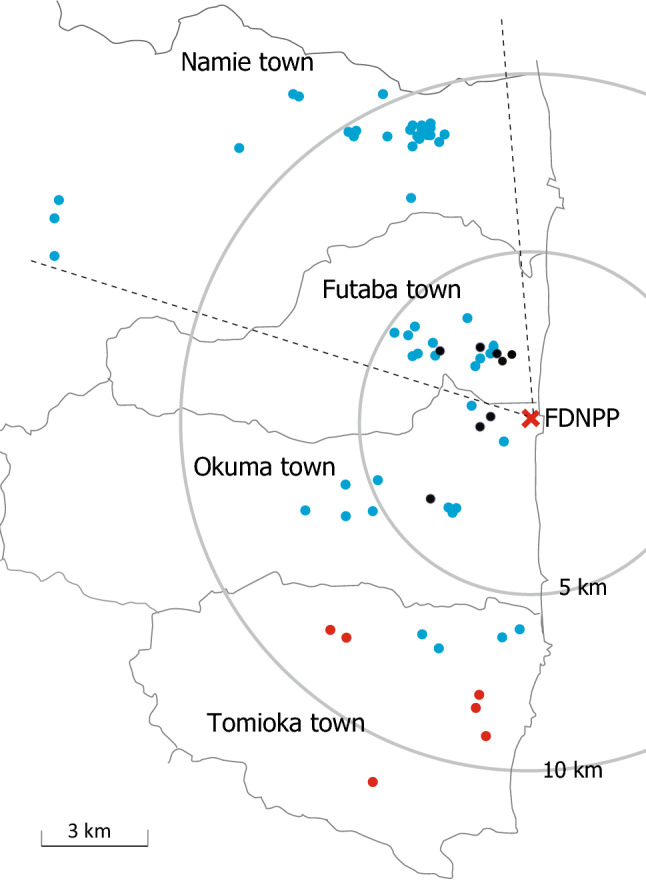
Map of the measurement locations, created using Adobe illustrator software (version 24.2.3).

**Figure 2:**
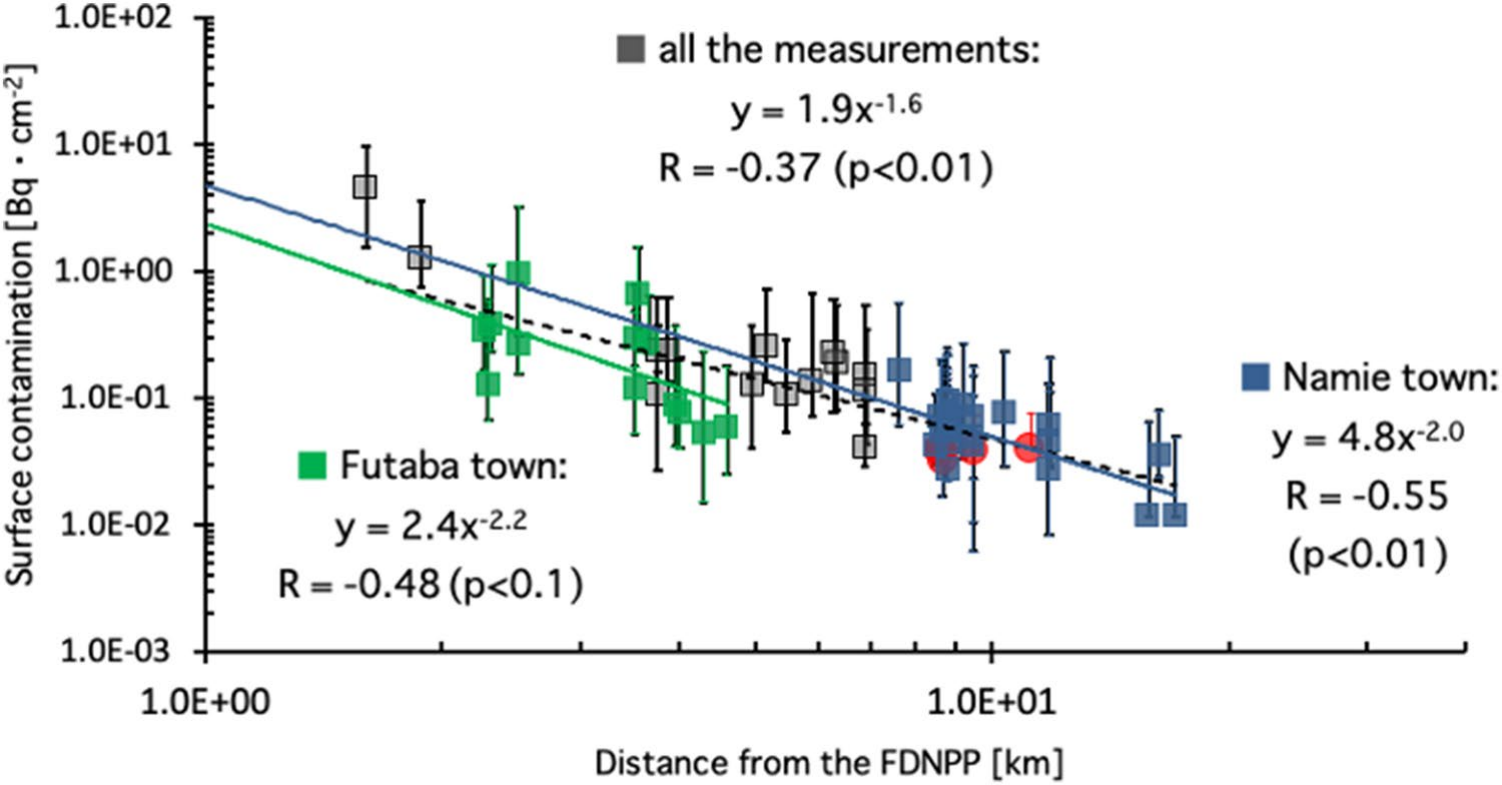
Median ^137^Cs surface contamination (as of March 2011) with an interquartile range of Q1–Q3 for each house as a function of distance between each house and the FDNPP. All the measurements from the 57 residential houses are shown. Red circles denote the inhabited houses whereas the others were uninhabited. The measurements for the 12 houses in Futaba town and the 25 houses in Namie town are exhibited as green and blue squares, respectively.

The locations of the 63 houses and two buildings investigated in the towns of Namie, Futaba, Okuma, and Tomioka, Fukushima Prefecture are shown in Fig. 1 as blue, black, and red circles. The locations of the houses and buildings and three colors of circles in Fig. 1 are shown in in Supplementary Table S1. One circle indicates one house or building investigated at each location. Six red circles in Tomioka town denote the inhabited houses, whereas blue and black circles denote the uninhabited houses. A total number of 2666 smear samples were collected from 57 residential houses that are shown as blue and red circles. The differences of each color of circle in Fig. 1 are shown in Supplementary Table S2. In Fig. 2, the median ^137^Cs surface contamination (as of March 2011) with an interquartile range of Q1–Q3, which are the middle values in the first and the second halves of the rank-ordered data set, for each house were plotted as a function of the distance between each house and the FDNPP. All the measurements from 57 residential houses were exhibited in Fig. 2, in which the red circles denote the inhabited houses, whereas others are uninhabited. The measurements for the 12 houses in Futaba town and 25 houses in Namie are shown as green and blue squares, respectively. Equations by power approximation are shown in Fig. 2, indicating that surface contamination is inversely proportional to the square of the distance between a house and the FDNPP for all curves. Specifically, R = − 0.37 (*p* < 0.01) for all the measurements, and R = − 0.48 (*p* < 0.1) and R = − 0.55 (*p* < 0.01) for the houses in the town of Futaba and Namie, respectively.

### The correlation between indoor surface contamination and air exchange rate

The air exchange rate for each house was measured using the CO_2_ decay method (the results are shown in Supplementary Fig. S1) and the median ^137^Cs surface contamination (as of March 2011) with an interquartile range of Q1–Q3 was plotted against the air exchange rate for each house in Fig. S2. No significant correlation was observed between surface contamination and air exchange rate. The values within red circles in Fig. S2 are from the reinforced concrete houses, in which three values on the left are from the 1st, 2nd, and 3rd floors of the apartment house showing nearly identical air exchange rates, whereas one value on the right from a two-storied house shows an air exchange rate three times larger than others. However, there was no difference among their four values of surface contamination. Alternatively, the wooden house within a blue circle was less ventilated, but shows the highest surface contamination among all. This house is the closest one (1.6 km distance) to the FDNPP. This result suggests that air exchange rate is unlikely correlated with surface contamination but the distance from the FDNPP and the initial dry deposition into the inside of the houses is considered to be dominant in surface contamination. If deposited dust in the uninhabited houses was resuspended in the indoor environment during through the years following the nuclear accident, resuspended aerosol can be exhausted to the outdoors by air exchange and remaining indoor radiocaesium would reduce largely in the houses with higher air exchange rates compared to those with lower air exchange rates. As shown in Fig. S2, no significant correlation between surface contamination and the air exchange rates was observed, indicating that the deposited dusts were not resuspended in the uninhabited houses. This is likely caused by the strong adsorption of surface contaminants on indoor surfaces. Furthermore, human activities such as walking can significantly increase the level of resuspended aerosols^13,14^, however, such human impacts are likely to be negligible given that the residents rarely returned and entered their houses during evacuation.

### Evaluation of aerosol resuspension factor from surface contamination

**Figure 3:**
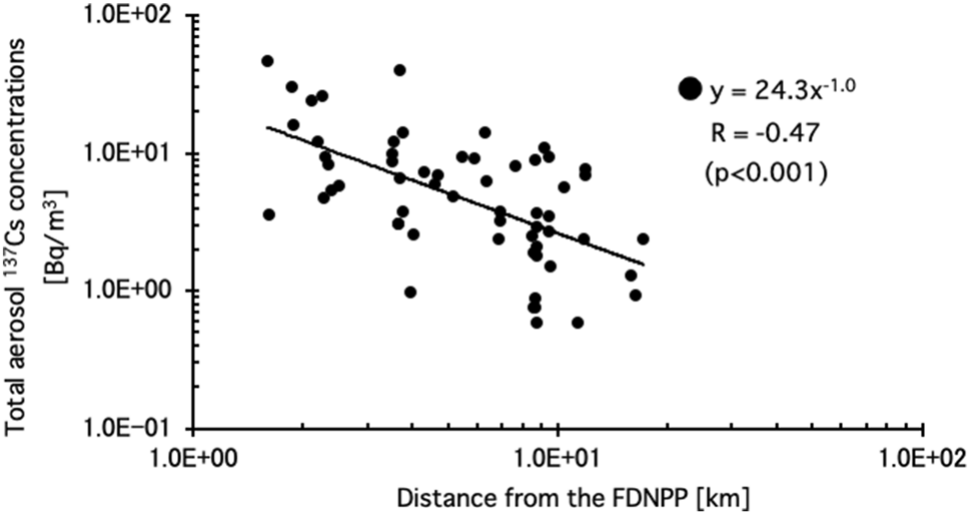
Correlation between total aerosol ^137^Cs concentrations (as of March 2011) in indoor air (the sum of the all particle sizes) during dusting is plotted as a function of distance from the FDNPP.

Correlations between the total aerosol ^137^Cs concentrations (as of March 2011) in indoor air (the sum of the all particle sizes) during dusting was plotted as a function of distance from the FDNPP in Fig. 3. The raw data set of the total aerosol ^137^Cs concentrations in indoor air during dusting was cited from the published Supplementary data in our previous report^12^. The result shows that the radioactivity concentration of ^137^Cs in indoor air during dusting was inversely proportional to distance between a house and the FDNPP [R = − 0.47 (*p* < 0.001)]. Data during the vacuuming with a cyclone cleaner and a normal paper pack type cleaner are not shown in Fig. 3, since the bulk of the measured ^137^Cs radioactivities during vacuuming for both types of cleaners were below the detection limit.

**Figure 4:**
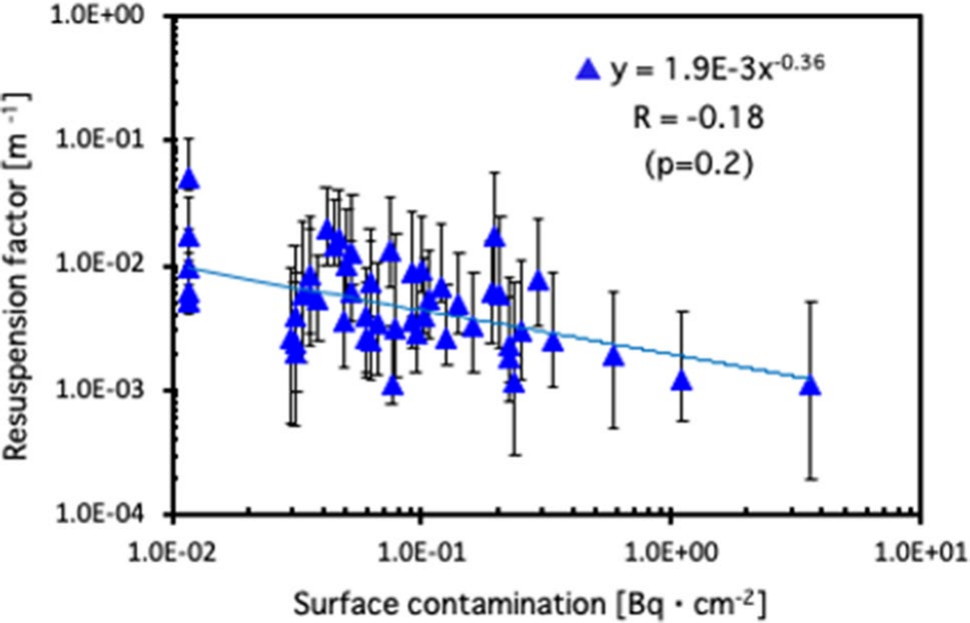
Resuspension factor for each house during dusting is plotted as a function of surface contamination.

Resuspension is described by a resuspension factor, *R* (m^−1^), which is the ratio of aerosol concentration at a reference height above a surface (Unit m^−3^) to the aerosol particle loading per unit area of the surface (Unit m^−2^)^15^. The European Model for Inhabited areas (ERMIN)^16^ defined outdoor re-suspension coefficient, *K* (m^−1^) as the ratio of the air concentration (mass per unit air volume) at a reference height above the surface to the concentration per unit area. Based on these definitions, the aerosol resuspension factor from surface contamination, *K* (m^−1^) for each house during dusting was defined by the following Eq. (1)^17^.

1 $$K = {\text{ CR}}_{{{\text{air}}}} / \, \left( {{\text{SC x 1}}0^{{4}} } \right)$$

where *K* is the resuspension factor (m^−1^), and CR_air_ is the radioactivity concentration of ^137^Cs in indoor air during dusting (Bq m^−3^), and SC is surface contamination (Bq cm^−2^). For CR_air_, the total aerosol ^137^Cs concentrations in indoor air were used and for SC, the median values with an interquartile range of Q1–Q3 of surface contamination of ^137^Cs were used. The raw data set of the total aerosol ^137^Cs concentrations in indoor air were cited from the published Supplementary data in our previous report^12^. The ^137^Cs values corresponded to the date of measurement of the aerosol samples. The resuspension factor, K for each house during dusting (N = 51) was plotted as a function of surface contamination in Fig. 4. No significant correlation between the resuspension factor and surface contamination was seen [Fig. 4; Pearson’s correlation coefficients: R = − 0.21 (*P* = 0.2)]. The average resuspension factor with one standard deviation was evaluated as 6.95 × 10^–3^ ± 7.80 × 10^–3^.

### The estimate of the internal exposure

**Figure 5:**
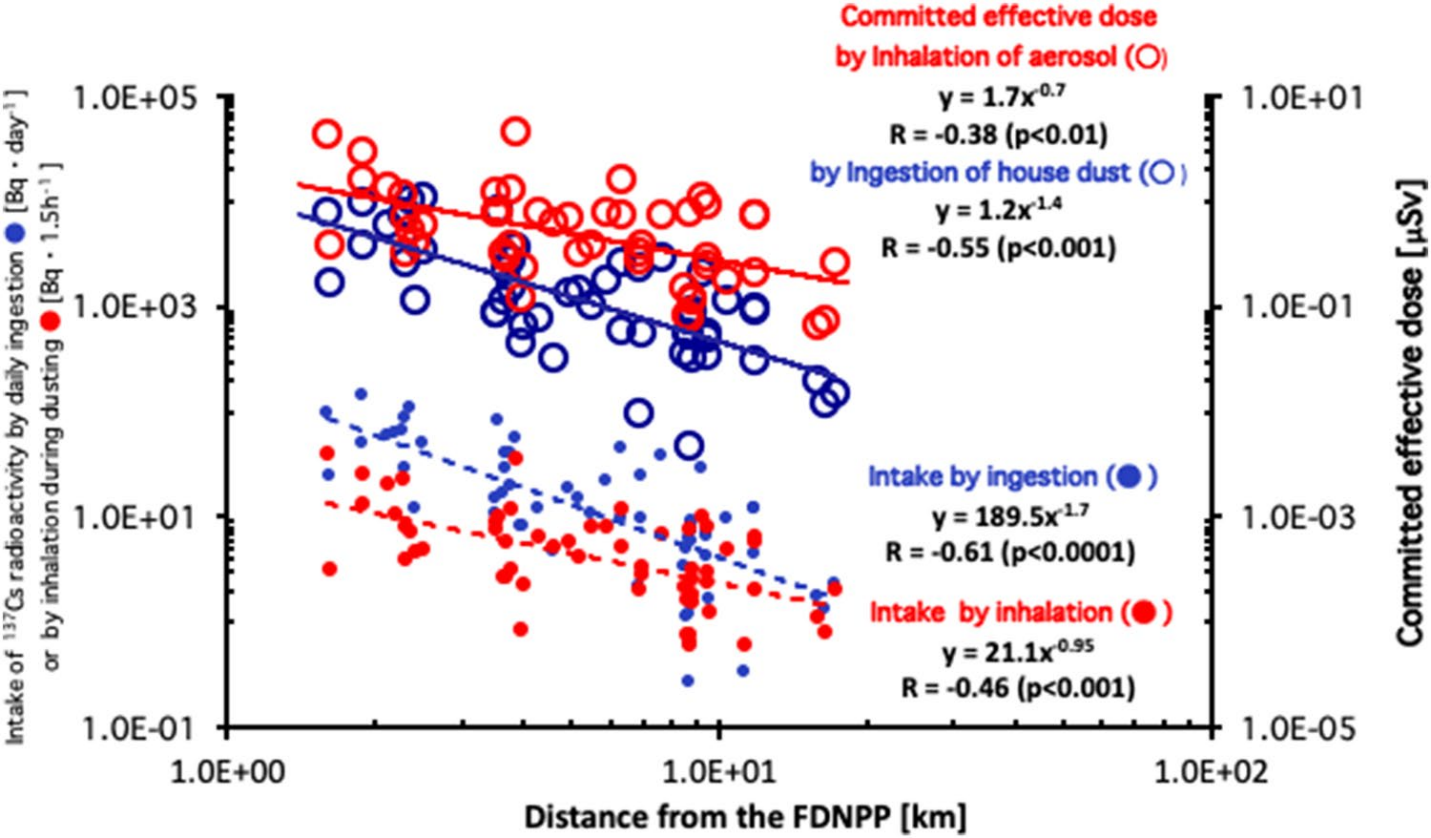
Daily ingested ^137^Cs radioactivity, I_d_ (filled blue circle) and the intake of radioactivity of ^137^Cs by inhalation, I_i_ (filled red circle) (as of March 2011) for each house is plotted as a function of distance from the FDNPP. The correlations between the committed effective doses owing to ingestion of the house dust (open blue circle) and those owing to inhalation of aerosol during dusting (open red circle), and distance from the FDNPP.

**Figure 6:**
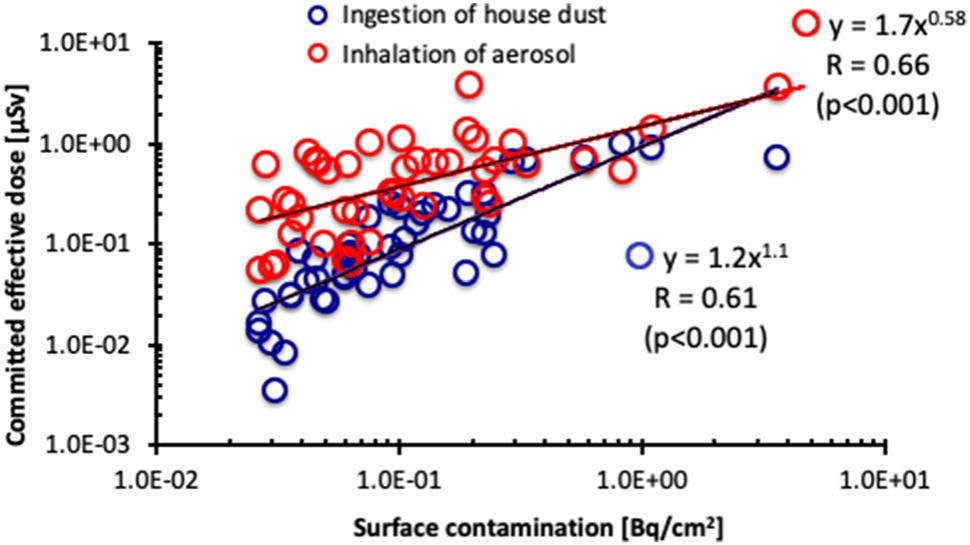
Correlations between the committed effective doses due to ingestion of the house dust (open blue circle) and those due to inhalation of aerosol during dusting (open red circle), and surface contamination.

The daily ingested ^137^Cs radioactivity, I_d_ (filled blue circle) obtained by Eq. (7) and the intake of radioactivity of ^137^Cs by inhalation, I_i_ (filled red circle) from Eq. (2) (Eqs. (7) and (2) are described in “Methods” section and the “Discussion” section, respectively), as of March 2011, for each house, was plotted as a function of distance from the FDNPP in Fig. 5 that display an inverse correlation in both curves, viz., R = − 0.61 (*p* < 0.0001) and R = − 0.46, (*p* < 0.001), respectively. The correlations between the committed effective doses, which were evaluated by using Cs^137^ radioactivities, as of March 2011, owing to ingestion of house dust (open blue circle), inhalation of aerosol during dusting (open red circle), and distance from the FDNPP, were shown in Fig. 5. Both the committed effective doses owing to ingestion of house dust and inhalation of aerosol were inversely correlated with distance from the FDNPP, viz., R = − 0.55 (*p* < 0.001) and R = − 0.38 (*p* < 0.01), respectively. The correlations between the committed effective doses owing to ingestion of house dust (open blue circle) and inhalation of aerosol during dusting (open red circle) and surface contamination, are shown in Fig. 6, in which the measured ^137^Cs values corresponded to the date of measurement of the aerosol samples. Surface contamination was clearly correlated with the committed effective doses from ingestion of house dust and inhalation of aerosol, viz., R = 0.61 (*p* < 0.001) and R = 0.66 (*p* < 0.001), respectively.

## Discussion

Inverse correlations between indoor surface contamination and the square of distance from the FDNPP in Fig. 2 are consistent with the Gaussian puff model^18^ that helps to predict the dispersion of the air pollution in the atmosphere.

At this stage, no clear difference in surface contamination was observed between the uninhabited (grey, green, and blue squares) and the inhabited houses (red circle) in the results in Fig. 2. Human activities such as walking and housecleaning can significantly increase the level of resuspended aerosols^13,14^, leading to a lower surface contamination with the air exchange in inhabited houses. Among the six inhabited houses in Fig. 2, there was only one house, in which samples were collected twice during the time of being uninhabited (January in 2015) and after residents resided in the house (December in 2018). For each collected data, 29% and 88% of them were below the detection limit. This house is located at 11.2 km from the FDNPP, which is rather far away from the FDNPP. Hence initial dry deposition for this house was quite low. The values of median ^137^Cs surface contamination (as of March 2011) with an interquartile range of Q1–Q3 were 0.046 (0.018–0.061) and 0.039 (0.036–0.039), respectively. The result of the Mann–Whitney U test shows a statistically significant difference (*p* < 0.001) between the two datasets. However, only one case is insufficient to discuss the effect of dwelling on indoor contamination. More measurements examples before and after residents reside in the house, which is closer to the FDNPP (having higher surface contamination), should be required to reveal the changes of surface contamination subsequent to the residents occupying the house. Further studies are also necessary to investigate the impact of residence time for uninhabited houses on surface contamination.

In Fig. 2, the measurements for houses in Futaba Town (green square) were compared to those for houses in Namie Town (blue square). The towns of Futaba and Namie are located in the same direction from the FDNPP, as shown by dotted lines in Fig. 1. Figure 2 shows the inverse correlations between surface contamination and the square of distance between a house and the FDNPP in both Futaba and Namie areas. The figure also shows a Y-intercept that was obtained by extending the curve for houses in Namie Town (4.8) that is twice higher than that for the houses in Futaba Town (2.4). The Tokyo Electric Power Company has estimated the amount of the radioactive materials and the trajectory for each radioactive plume released from the FDNPP based on the data of monitoring posts and meteorological data^19^. The estimations show that the radioactive plume released from Unit 1 on March 12, 2011 and the one from Unit 2 on March 15, 2011, passed over Namie Town, but not Futaba Town. Further, the radioactive plume with the largest amount of radioactivities that was released from Unit 2 on March 14, 2011 passed over Namie Town twice, whereas it passed over Futaba Town once, owing to the change of the wind direction. The result in Fig. 2 may have reflected these events.

Figure 3 shows that the total aerosol ^137^Cs concentrations during dusting inversely correlated with distance from the FDNPP. Dusting increased the aerosol ^137^Cs concentrations by brushing fine dust from the surface of walls, shelves, curtains, shoji screens (paper sliding doors), furniture, and clothes with a cotton fabric duster for 1.5–2.5 h^12^. In the previous study on indoor contamination^6^, we showed the same tendency that surface contamination was inversely proportional to the square of distance between a house and the FDNPP for houses close to the FDNPP (~ 12.0 km). These results imply a correlation between the total aerosol ^137^Cs concentrations during dusting and surface contamination, since aerosols are attributed to surface contamination.

The resuspension factors for different types of surfaces^15^ and resuspension rates for light activity in the residence^13^ were obtained from the ratio of aerosol concentration (gm^−3^) to the aerosol particle loading per unit area of the surface (gm^−2^), since they are not derived from radiation-related studies. The radiation-related research of the aerosol resuspension factor, which is determined by the ratio of the radioactivity concentration (Bq m^−3^) to surface contamination (Bq cm^−2^) by Eq. (1), was hitherto conducted mainly from the perspective of radiation safety control in nuclear facilities^17^, since air pollution due to the resuspension of radioactive contaminants such as plutonium can cause an unexpected internal exposure for radiation workers. There are no published data evaluating the resuspension factor during the actual cleaning within the affected houses following the nuclear accident. According to the survey results, which is obtained from the data measured during various radiation operations in institutions such as the Reprocessing Plant of Tokai Works, Power Reactor and Nuclear Fuel Development Corporation and Tokai and Oarai Research and Development Institute of Japan Atomic Energy Agency, the resuspension factor ranges on average from 1.0 × 10^–7^ to 1.0 × 10^–5^ cm^−1^ and from 1.0 × 10^–7^ to 1.0 × 10^–6^ cm^−1^ during the works on decontamination and repairment of equipment, and during the handling of plutonium, etc., respectively^17^. The result of resuspension factor, in this study, of 6.95 × 10^–3^ ± 7.80 × 10^–3^ cm^−1^ during dusting within the affected houses is two orders of magnitude greater than those in a nuclear facility. This difference is considered to have attributed to the differences in the type of work and in the conditions owing to the floor and wall/ceiling materials, including the presence of furniture between nuclear facilities and ordinary residential houses. Further, this was due to surface contamination, viz., a denominator in Eq. (1), for residential houses that might be underestimated since the values of surface contamination were determined by wiping the flat, smooth, and non-porus surfaces of wood, metal, glass, and plastic structures, while excluding cloths or friable materials such as carpets, blankets or futons, since the removal fraction, F^6^, cannot be determined for the latter.

The amount of ^137^Cs intake for two workers of A and B involved in dusting within the houses in Futaba town were measured by (a) a whole-body counter, WBC, and compared with the values evaluated from (b) ^137^Cs radioactivity concentrations in indoor air. As for the method (a), the ^137^Cs intake was measured for 30 min for each person on June 27, 2017 at National Institutes for Quantum and Radiological Science and Technology, using a precision-type WBC, which has six HPGe detectors that are installed in a large shielded chamber made of 20 cm-thick-steel^20^. The measurement with a WBC yielded 40.1 Bq for worker A, and a level below the minimum detectable amount, MDA, which was estimated as 18.5 Bq for worker B. In the method (b), the radioactivity of ^137^Cs intake from inhalation of aerosol, *I*_i_ was calculated using the equation:

2 $$I_{{\text{i}}} = {\text{ CR}}_{{{\text{dusting}}}} \times {\text{ RE }} \times {\text{ t }} \times {\text{ PF}}^{ - 1}$$

where CR_dusting_ is the total radioactivity concentration of ^137^Cs for all diameters of aerosols (< 0.25, 0.25–0.50, 0.50–1.0, 1.0–2.5, 2.5–6.6, and > 6.6 μm) in air during dusting (Bq m^−3^), RE, the respiratory rate (m^3^ h^−1^), t, the time (h) and PF, the protection factor of the mask. For the respiratory rate, RE, the value of 1.17 (m^3^ h^−1^) was used as the average respiratory rate of Japanese men and women during doing housework^21^.

For worker A, within three houses of a, b, and c, where worker A was engaged in dusting, the measured values of CR_dusting_ were 5.0 (the main building of house a), 61.2 (detached room of house a), 23 (house b), and 5.2 (house c) (Bq m^−3^) as of each measurement date, respectively. Actual working times were 140, 37, 119, and 129 min, respectively. Total intake of radioactivity of ^137^Cs, *I*_i_ was calculated as 97.7 Bq. The effective half-life of ^137^Cs of 110 days was taken into account based on the physical half-life of 30.07 years and the biological half-life of 110 days^22^ since there are elapsed days between the working date and the measurement date with a WBC. The same calculation of total intake of radioactivity of ^137^Cs, *I*_i_ for worker B was conducted, resulting in 12.0 Bq. The results for workers A and B are summarized in Table 1. Comparing the calculated results (97.7 Bq) and the measurement with a WBC (40.1 Bq) for worker A, the protection factor, PF of the mask, was set as 2 and this value was used as PF in this study. Worker A had eaten a river fish and mountain vegetables for supper in Kawauchi village 21 days before the measurement with a WBC, and whose radioactivities were not examined. However, it is unlikely to happen that the significant radioactivity of ^137^Cs was detected that exceeds the MDA of a WBC owing to a meal at one time, since the radioactivity of ^137^Cs detected from fish and vegetables growing naturally in mountains and fields were declining with time^23^. Hence, we have assumed that the radioactivity of ^137^Cs detected from worker A was attributed to dusting. The results in Table 1 indicate that the calculated results from ^137^Cs radioactivity concentrations in indoor air and the measurement with a WBC are consistent for the two cases of workers A and B. Note that in an actual life, where opening the windows and doors of the room is a common practice during cleaning, it is unlikely that the intake of radioactivity of ^137^Cs during dusting will be as high as the value in Table 1, where dusting was conducted with the windows and doors of the room closed. Furthermore, it is unlikely that ^137^Cs that exceeds the MDA of a stand-up geometry WBC (FASTSCAN manufactured by Canberra), which is commonly used to monitor internal exposure of an affected resident, will be detected from the residents who dust the rooms, since the MDA is evaluated as 300 Bq^24^ for ^137^Cs, when measured with a typical count time of 2 min employing a FASTSCAN.

**Table 1:** Intake of radioactivity of ^137^Cs by calculating from ^137^Cs radioactivity concentrations in indoor air and one by measuring with a WBC.

Worker	^137^Cs calculated from radioactivity concentrations in indoor air	^137^Cs measured with a WBC		
		Activity (Bq)	MDA (Bq)	Error (%)
A	97.7	40.1	24.5	12.89
B	12.0	> 15.232	18.5	–

The moderate inverse correlations between both the committed effective dose from ingestion of house dust (open blue circle) and those (open red circle) from inhalation of aerosol with distance from the FDNPP (Fig. 5) indicates that the internal exposure doses from the remaining radioactive Cs within the house for returnees can be roughly estimated when distance from the returnee’s house to the FDNPP is known. When both the committed effective doses are plotted as a function of surface contamination, the results clearly exhibit positive correlations (Fig. 6) with a better coefficient, R for both the committed effective doses from ingestion of house dust and from inhalation of aerosol with surface contamination (0.66 and 0.61, respectively) than those between the committed effective doses and distance from the FDNPP (− 0.55 and − 0.38, respectively; Fig. 5). This result suggests that surface contamination can be utilized by a useful marker for estimating the committed effective dose from ingestion and inhalation. Sampling with the smear method is simple and convenient, and requires a short time for collecting the samples, compared to sampling by cleaning by vacuuming and dusting.

Apart from the house dust and aerosol, it should be noted that the non-spherical heterogeneous radiocaesium-bearing particles were found on masks worn during cleaning in this study^25^. A stochastic biokinetic method was developed to evaluate the probability density function of internal doses for inhalation of a particulate material^26^, assuming that these particles are insoluble. However, the characteristics of these particles such as thermal and dissolution properties are under investigation^27^. Further study is required to estimate the internal dose due to inhalation of radiocaesium-bearing particles.

The highest committed effective doses from ingestion and from inhalation, as shown in Fig. 5, were 1.13 µSv per a day (for the house 2.50 km from the FDNPP) and 4.55 µSv during dusting (for another house 3.87 km from the FDNPP), respectively. These two houses are located within the designated reconstruction and rehabilitation base areas^4^ in the towns of Futaba and Okuma, respectively. In these areas, the evacuation issues are to be lifted and the residents will be allowed to return and live within 5 years. Assuming that the residents in each of the two houses restart their lives on Jan 1 2023, their committed effective doses by daily intake of the house dust and by inhalation of aerosol during dusting once a week, *E*(t_1_,t_2_)_ingestion_ and *E*(t_1_,t_2_)_inhalation_, respectively, during the period between t_1_ (set as Jan 1 2023) and t_2_ are obtained by the following equations,

3 $$E\left( {{\text{t}}_{{1}} ,{\text{t}}_{{2}} } \right)_{{{\text{ingestion}}}} = \mathop \int \limits_{{t_{1} }}^{{t_{2} }} E\left( t \right)_{ingestion} \cdot d_{t}$$

4 $$E\left( {{\text{t}}_{{1}} ,{\text{t}}_{{2}} } \right)_{{{\text{inhalation}}}} = \mathop \int \limits_{{t_{1} }}^{{t_{2} }} E\left( t \right)_{inhalation} \cdot d_{t}$$

where *E*(t)_ingestion_ and *E*(t)_inhalation_ are the highest committed effective doses by ingestion of house dust and by inhalation of aerosol at an elapsed time t (µSv), obtained from Eqs. (5) and (8) that are described in the Method section, respectively.

The calculated results are shown in Supplementary Fig. S3. However, the results might be overestimated since the residents commonly open the windows and doors of the room during cleaning, whereas every door and window was kept closed during the collection of aerosol sample for this study, and the indoor contamination can be removed by the cleaning.

**Figure 7:**
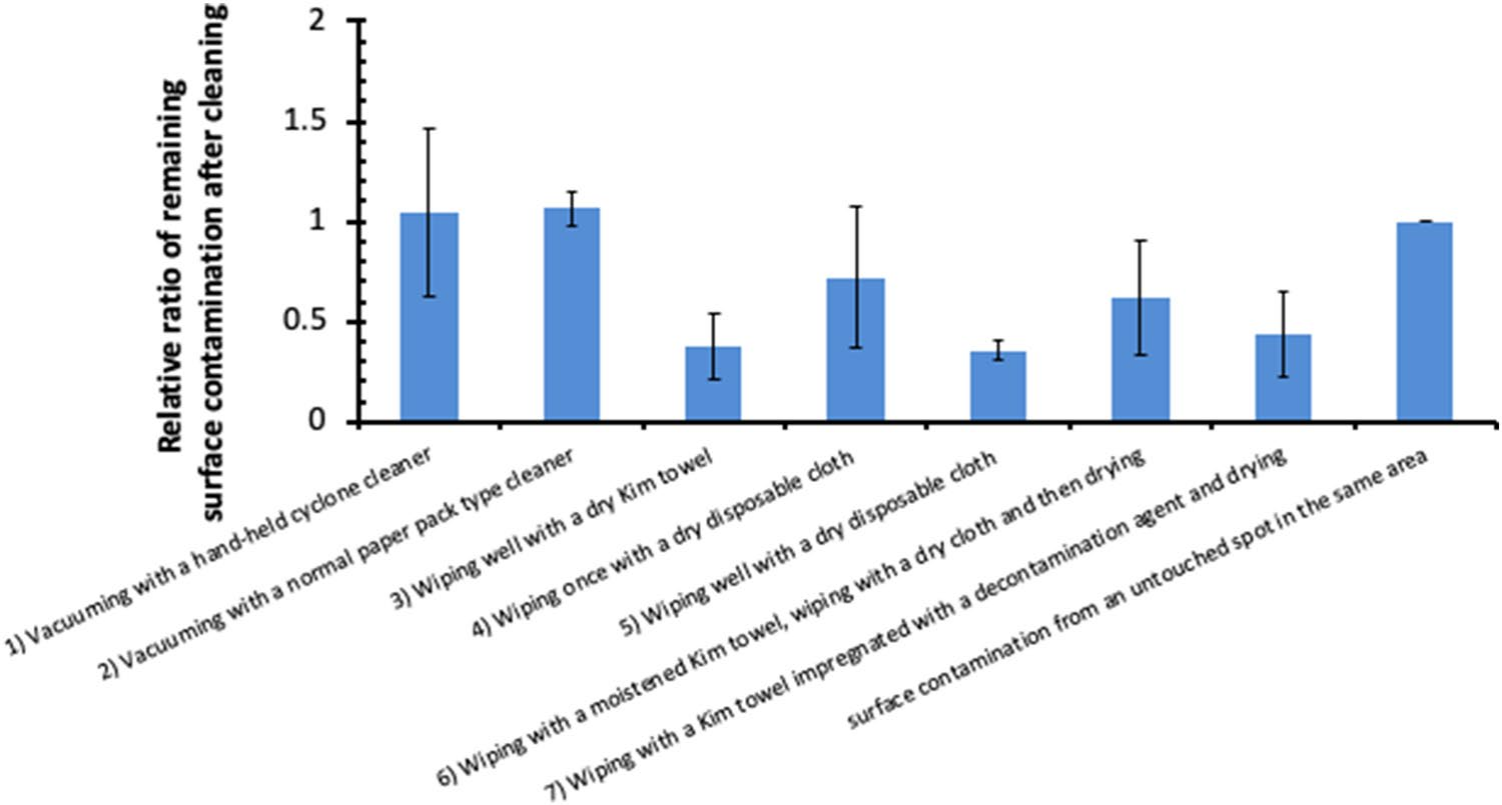
Relative value of the remaining surface contamination after applying different removal (cleaning) methods to the radiocaesium contamination attached to the surface. Each value is standardized with that of the surface contamination from an untouched spot in the same area.

To reduce the internal exposure after residents make a permanent return to their home, tips for the effective removal of the remaining radioactivity within the house is required. We have examined seven different types of method (dry and moistened method) to remove radiocaesium surface contamination in 10 houses having a smooth flat area sufficient to allow multiple smear sampling in difficult-to-return areas, where surface contamination is relatively high, within Okuma Town, Tomioka Town, and Namie Town. The examined methods are as follows; (1) vacuuming with a hand-held cyclone cleaner, (2) vacuuming with a normal paper pack type cleaner, (3) wiping well with a dry Kim towel, (4) wiping once with a dry disposable cloth, (5) wiping well with a dry disposable cloth, (6) wiping with a moistened Kim towel, wiping with a dry cloth and then drying, and (7) wiping with a Kim towel impregnated with a decontamination agent and drying. Subsequent to each method being applied to a different area grid of approximately 15 cm × 15 cm area (N = 190 in total) on the same area, a dry smear test was applied to the area grid of 100 cm^2^ of the surface inside of each area. In Fig. 7, the results of the remaining surface contamination after applying each removal method to radiocaesium contamination attached to the surface are shown by standardizing them with the value of surface contamination from an untouched spot in the same area, assuming that the initial value of surface contamination is the same in the examined area. In Fig. 7, each column and bar denote the average value and 1σ for each removal method. In the results, after applying removal methods of (3), (5), and (7), all the values plummeted below the detection limit. For these values, half of the detection limit was used for calculating the means and SDs. Note that the results were greater than 1 after applying removal methods (1) and (2). This result suggests that surface contamination still remained after vacuuming with a cyclone cleaner or a normal paper pack type cleaner. Further, it also implies that surface contamination was lifted from the surface by vacuuming to allow surface contamination to be easily removed by a dry smear test. Lifted surface contamination from the surface has increased the value of removal fraction, F, which is explained in Supplementary Methods, resulting in the apparent increase in surface contamination in methods (1) and (2). The results in Fig. 7 also suggest that three methods of (3), (5), and (7) were extremely effective in removing surface contamination and these should be recommended as the cleaning methods to avoid internal exposure, since they do not cause resuspension of aerosol unlike dusting. It was emphasized that a cyclone cleaner and a normal paper pack type cleaner can vacuum house dust, but cannot remove surface contamination which is firmly attached to the surface. The remaining contamination after method (4) exceeded that after methods (3) or (5), indicating that the wiping of the surface with a Kim towel or a cloth just once is insufficient to fully remove contamination. Further, a thorough wiping the surface is required since radiocaesium contamination must have attached to the surface firmly.

## Methods

### Locations of the houses and buildings investigated

During the period from April 2016 to January 2019, house dust and aerosols were sampled from 65 houses and buildings in total in the towns of Namie, Futaba, Okuma, and Tomioka, Fukushima Prefecture (Fig. 1, as shown in blue, black, and red circles; Table S1), where all the administrative districts were designated as evacuation areas in 2011 after the FDNPP accident. Of the 65 houses, 51 are made of wood (49 detached residential houses and two wooden community centers), 10 of light-gauge steel, and four of reinforced concrete (one detached residential house and three apartment residential houses). All such houses are single and (or) two-storey structures except three residential apartment houses. During the investigation, 26 houses within the towns of Namie and Tomioka were in the areas where the evacuation order was lifted. Of these 26 houses, the residents returned to their homes in six houses in Tomioka Town (red circles in Fig. 1 and Table S2). All the houses and buildings except the six houses had remained uninhabited.

### Indoor surface contamination sampling

Total number of 2666 smear samples were collected from 52 residential houses (51 samples per house on average), shown as blue and red circles in Fig. 1. A size of 2465 samples were collected from 46 houses during a period from April 2016 to January 2019, whereas 201 samples from 6 houses were collected during a period from November 2014 to January 2015.

A dry smear test^28,29^ was applied to the surface of the materials and structures in rooms. The details corresponding to the method of collecting the smear samples is described in Supplementary Methods. In Supplementary Fig. S4, a typical example of a room investigated is shown with smear sampling positions.

Methods on the cleaning, house dust sampling, aerosol sampling, separation depending on extractability, and air exchange rate are described in Supplementary Methods.

### Determination of ^137^Cs radioactivity

The ^137^Cs radioactivity of samples was determined using a high-purity germanium (HPGe) detector with a relative efficiency of 33.5% (EGPC 50-195-R, Eurisys, France). The data were analyzed by Gamma Studio (SEIKO EG&G)^10,12^. The detection efficiency was determined using a certified reference source of ^137^Cs (Amersham International plc, Buckinghamshire, UK). The full-energy peak efficiency was 0.6328% for a peak energy of 662 keV (^137^Cs). The measured activity was checked by periodically comparing with the certified ^137^Cs activity of the reference source, and the differences between them were evaluated within 3.5%. The accuracy of the reference point source of ^137^Cs, which is defined as the overall uncertainty of the result, is guaranteed as 4%. The counting time for each sample was 1000–10,000 s.

The ^137^Cs source is a point source and the shape and size of the samples differ. The details of the evaluation of the differences in the detection efficiency between the certified radioactivity and the samples are described in Supplementary Methods.

To save the occupancy time of the HPGe detector, we measured the radioactivity on the smear and aerosol samples using beta rays emitted from ^137^Cs for 10 min and 100 min, respectively, with a plastic scintillator detector JDC-5300 (Hitachi Aloka Medical, Ltd., Japan). Determination of the ^137^Cs radioactivity for the smear and aerosol samples is described in Supplementary Methods.

Determination of ^137^Cs radioactivity for solution sample extracted from the dust and for indoor contaminants is described in Supplementary Methods.

### Estimation of the internal exposure

The committed effective doses were calculated by DCAL (Dose and Risk Calculation System) software^30^. DCAL, which is an internal exposure dose evaluation software system, performs biokinetic and dosimetric calculations for the case of acute intake of a radionuclide by ingestion and inhalation. DCAL is designed to allow expansion of its library of gastrointestinal uptake values (f_1_ values) with user-supplied models.

Internal exposure by ingestion of the house dust was estimated as follows. The relative ratio of fractions extracted in water, in 1 M HCl, and the residue fraction, which are insoluble to water and 1 M HCl, were obtained for each particle size for the house dust in each house. Each fraction that is extracted in water and in 1 M HCl, is regarded as that soluble to water and to 1 M HCl, respectively. Dose coefficients (Sv Bq^−1^) for the intake of ^137^Cs by adults, e_ingestion,f1=1 _ and e_ingestion,f1=0.1 _ were 1.36 × 10^–8^ and 2.55 × 10^–9^, respectively, as measured by DCAL by adopting an f_1_ value of 1 and 0.1 for the fractions soluble to water or 1 M HCl and the insoluble residue fraction. The values of f_1_ follow ICRP Publ. 137, in which an f_1_ value of 1 is adopted for all forms of caesium, except in situations where it is considered that the material is insoluble and a lower f_1_ value of 0.1 is appropriate^31^.

The committed effective doses by ingestion of house dust, *E*_ingestion_ (µSv) were obtained using the following equation.

5 $$E_{{{\text{ingestion}}}} = {\text{ I}}_{{\text{d}}} \times \left( {e_{{ingestion, f_{1} = 1}} \times M_{soluble} + e_{{ingestion, f_{1} = 0.1}} \times M_{insoluble} } \right) \times 10^{ - 6}$$

where I_d_ is the daily ingested ^137^Cs radioactivity (Bq), and M_soluble_ and M_insoluble_ are the relative ratios of water or 1 M HCl soluble fraction to total, and insoluble (the residue) fraction to total, respectively. The sum of M_soluble_ and M_insoluble_ equals one. The value of M_soluble_ for each house against distance from the FDNPP is shown in Supplementary Fig. S8.

The daily ingested ^137^Cs radioactivity, I_d_ was obtained as follows. Internal exposure from ingestion is considered to attribute to the house dust adhered to one’s hands through the hand-to-mouth route. Yamamoto et al.^32^ revealed that the larger soil particles of approximately 200–300 μm rarely adhere to hands, whereas the soil particles of 39 ± 26 μm do adhere. Edwards and Lioy have indicated that the particles < 100–200 μm in size were retained most efficiently by skin^33^. Based on these findings, we have used portions of the house dust of < 4–180 μm from the collected samples by particle size (< 4‒180, < 4‒20, 20‒63, 63‒180, 180‒500, 500‒1000, and 1000‒2000 μm) for calculation of internal exposure from ingestion. The ^137^Cs radioactivity concentration of < 4‒180 μm portions of house dust, viz. C_<4‒180_ (Bq g^−1^) was obtained for the house dust in each house as the dust-weight-weighted average of each size fraction using the following equation.

6 $$C_{ < 4 - 180} = \frac{{\mathop \sum \nolimits_{d = < 4 - 20}^{63 - 180} C_{d} \times W_{d} }}{{\mathop \sum \nolimits_{ < 4 - 20}^{63 - 180} W_{d} }}$$

where C_d_ (Bq g^−1^) is ^137^Cs radioactivity concentration in the house dust in each size fraction d and W_d_ (g) is dust weight for each size fraction d. The raw data set of ^137^Cs radioactivity concentration in the house dust in each size fraction and dust weight for each size fraction were cited from the Supplementary data published in our previous report^12^.

Thereafter, the daily ingested ^137^Cs radioactivity, I_d_ (Bq) was obtained using the following equation.

7 $$I_{d} = C_{ < 4 - 180} \times I_{house dust } \times 10^{ - 3}$$

where I_house dust_ is the daily intake of the house dust, and the recommended value of 20 (mg day^−1^) for adults^34^ is adopted.

Internal exposure by inhalation of aerosol was estimated as follows. Aerosol that was resuspended during dusting is considered as the cause of the internal exposure since almost all measured ^137^Cs radioactivities during the vacuuming for both types of cleaners were below the detection limit. For exposures in the general environment, 1-µm median aerodynamic diameter (AMAD) is considered as the appropriate value, by assuming a lognormal distribution for the particle size distribution^8^. However, our previous study revealed that the ^137^Cs radioactivity concentration in aerosols increases with the aerodynamic diameters of the particles, and hence the distribution of aerosol particle aerodynamic diameters does not show a logarithmic normal distribution^12^. Taking this finding into consideration on the safe side, we have replaced the deposition rate for each compartment with the maximum value among all compartments and calculated the committed effective doses by DCAL^30^. We have assumed that the solubility of the aerosol is the same as that of the house dust based on the suggestion that indoor ^137^Cs contamination measured in aerosols during dusting originated from the smaller particles of the indoor house dust^12^. Dose coefficients (Sv Bq^−1^) by inhalation, e_inhalation,f1=1_ and e_inhalation,f1=0.1_ of ^137^Cs for adults were obtained by DCAL as 1.800 × 10^–8^ and 1.918 × 10^–7^, respectively, by adopting an f_1_ value of 1 and 0.1 for the house dust components extracted in water or 1 M HCl and the residue component^10^, respectively.

The committed effective doses by inhalation of aerosol, *E*_inhalation_ (µSv) were obtained using the following equation.

8 $$E_{{{\text{inhalation}}}} = {\text{ I}}_{{\text{i}}} \times \left( {e_{{inhalation,f_{1} = 1}} \times M_{soluble} + e_{{inhalation,f_{1} = 0.1}} \times M_{insoluble} } \right) \times 10^{ - 6}$$

where I_i_ is an intake of radioactivity of ^137^Cs (Bq) by inhalation from resuspended aerosol during dusting, and M_soluble_ and M_insoluble_ are the same values used in Eq. (5).

The intake of radioactivity of ^137^Cs, I_i_ by inhalation of the resuspended aerosol during dusting was obtained by using Eq. (2) and 1.5 h for time period, t. PF was set to 2, and the reason for taking the value 2 for PF was described in the “Discussion” section. The raw data set for CR_dusting_ was cited from the Supplementary data published in our previous report^12^.

### Ethical approval

This study was approved by the ethics review committee concerning research with human subjects at the Graduate School of Pharmaceutical Sciences, Tohoku University, Japan. All experiments were performed in accordance with relevant guidelines and regulations. For the residents whose houses were examined in this study, the subject, object, sampling and measurement methods, and publication methods were explained verbally or in writing, and written informed consent was obtained from each resident prior to the examination.

## Supplementary information

Supplementary Information.
