# Supplementary material for: Estimated internal exposure doses due to indoor radiocaesium contamination in residential houses after the Fukushima nuclear accident
Source: Sci Rep. 2020 Oct 14;10:17212. doi: 10.1038/s41598-020-74182-x (PMC7560611; doi:10.1038/s41598-020-74182-x)
Supplement: Supplementary file 1 — Supplementary Information. [file 41598_2020_74182_MOESM1_ESM.doc]

Estimated internal exposure doses due to indoor radiocaesium contamination in residential houses after the Fukushima nuclear accident

Hiroko Yoshida-Ohuchi^*a^, Naohide Shinohara^b^

^a^Graduate School of Pharmaceutical Sciences, Tohoku University, 6-3 Aramaki-Aoba, Aoba-ku, Sendai, Miyagi 980-8578, Japan

^b^Research Institute of Science for Safety and Sustainability (RISS), National Institute of Advanced Industrial Science and Technology (AIST), 16-1 Onogawa, Tsukuba, Ibaraki 305-8569, Japan

Supplementary Information

**Table S1**. List of the locations of the 63 houses and 2 buildings and marks of blue, black, and red circles in Fig.1.

| Locations | Distance from the FDNPP | Houses and buildings | Marks |
| --- | --- | --- | --- |
| Namie town | 7.6–16.1 km | 25 houses | 25 blue circles |
| Futaba town | 2.1–4.6 km | 15 houses | 12 blue circles and  3 black circles |
|  |  | 2 buildings | 2 black circles |
| Okuma town | 1.6–6.9 km | 13 houses | 10 blue circles and  3 black circles |
| Tomioka town | 6.3–11.2 km | 10 houses | 4 blue circles and  6 red circles |

**Table S2**. List of the differences of each color of circle in Fig.1.

| Color of circle | Dry smear test | Dusting and vacuuming | Residence |
| --- | --- | --- | --- |
| Blue | ○ | ○ | remaining uninhabited |
| Black |  | ○ | remaining uninhabited |
| Red | ○ | ○ | inhabited |

The air exchange rate for each house was measured using the CO^2^ decay method.


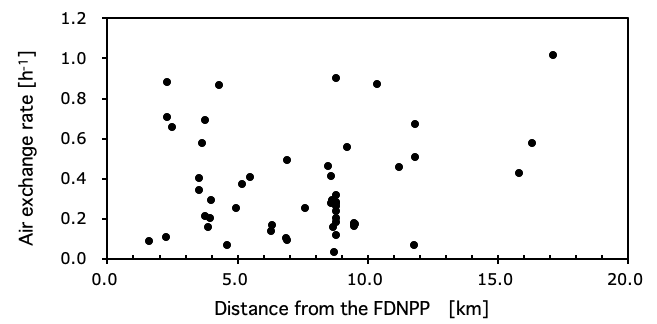


Fig. S1 Air exchange rate for each house against distance from the FDNPP.

Median surface contamination with an interquartile range of Q1–Q3 was plotted against air exchange rate for each house. No significant correlation was observed between surface contamination and air exchange rate.


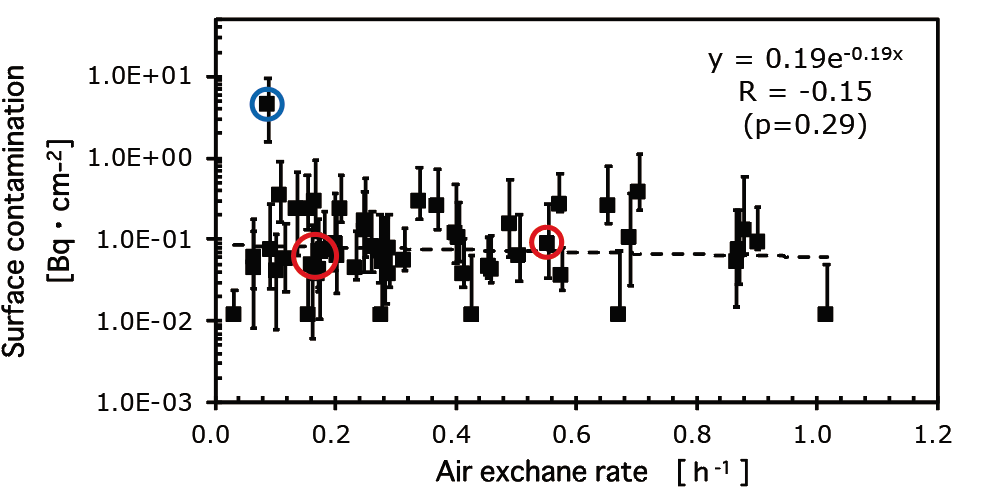


Fig. S2 Median ^137^Cs surface contamination (as of March 2011) with an interquartile range of Q1–Q3 against air exchange rate for each house.

Fig. S3 Committed effective doses by daily intake of the house dust and by inhalation of aerosol during dusting once a week, *E*(t_1_,t_2_)_ingestion_ and *E*(t_1_,t_2_)_inhalation_, respectively during the period between t_1_ (set as Jan 1 2023) and t_2_.

Methods

Indoor surface contamination sampling

An area of 100 cm^2^ of the surface from wood materials in most cases (from metal, glass, and plastic materials in a few cases) were rubbed with moderate pressure using a round smear test paper with a diameter of 25 mm^1)^. The smear samples were carefully collected from flat, smooth, and non-porus surfaces in every room. For all materials in the rooms, the horizontal surfaces were rubbed. Smear samples were collected before cleaning (vacuuming and dusting) was conducted. In certain houses, where large material disturbance such as soil, excretion, and small pieces of broken glass were found due to the intrusion of wild animals and (or) burglars, the smear samples were collected while avoiding the disturbance.


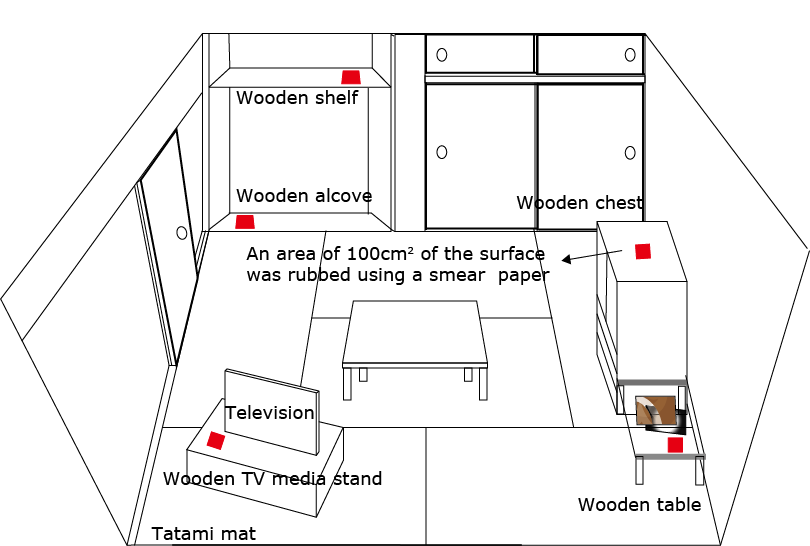


Fig.S4 Typical example of a room investigated with smear sampling positions, created using Adobe illustrator software (version 24.2.3).

Cleaning method

A hand-held cyclone cleaner (DC61MH; Dyson Ltd., UK) for all houses investigated and a normal paper pack type cleaner (VC-PF7A (L); Toshiba Lifestyle Products & Services Co. Ltd., Japan) for 14 houses where electric supply was restored, were used for vacuuming and sampling of the house dust. Then, dusting of the surface of wall, shelf, and furniture was followed using a cotton fabric duster (Taiyo-cotton duster 52012; Yatsuya Kogyo Co,.Ltd, Japan) for 1.5–2.5 h. Throughout the process of vacuuming and dusting, every door and window was kept closed, and no ventilation fan was operated. In some houses, vacuuming and dusting were simultaneously conducted.

House dust sampling

House dust was sampled for approximately 1.5–2.5 h by particle size using a cyclone vacuum (DC61MH; Dyson) with multistage sieves (20, 63, 180, 500, 1,000, and 2,000 µm) and a 70-mm polytetrafluoroethylene filter (pore size 4 µm, Advantec PF040, Toyo Roshi Kaisha, Ltd., Japan). In a laboratory, the house dust samples were additionally sieved by shaking with a mixer (VTX-3000L; LMS Co. Ltd., Japan) and weighing with a balance (AUW120D; Shimadzu Co., Japan).

Aerosol sampling

Aerosols with aerodynamic diameters of 0.25–0.50, 0.50–1.0, 1.0–2.5, 2.5–6.6, and >6.6 μm were sampled using polytetrafluoroethylene filters (25 mm φ, 0.5 μm pore size, 225-2708, SKC Inc., USA), and aerosols with aerodynamic diameters of <0.25 μm were sampled using polytetrafluoroethylene filters with support rings (37 mm φ, 0.5 μm pore size, 225-1709, SKC Inc., USA) at 9.0 L/min using a portable pump (Leland Legacy Pump, SKC Inc., USA) in a cascade impactor (Sioutas Cascade Impactor, SKC Inc., USA) during approximately 1.5–2.5 h of vacuuming or dusting. The sampling position was always set close to a person cleaning the room/corridor at a height of approximately 1.5 m in each room/corridor.

Particles could not be adequately trapped on the filter owing to the rebound from the surface of filter. Therefore, aerosols were sampled with two or four sets of pumps and impactors; in one or two of the impactors, the polytetrafluoroethylene filters were used with silicone grease (approximately 28.0 mg/filter), whereas the other one or two impactors used the filters without silicone grease.

Separation depending on extractability

House dust samples (50 or 100 mg) with 2.5 or 5.0 mL of pure water were centrifugally filtered at 12,000 × g for total of 120 min with polycarbonate ultrafiltration tubes (VIVASPIN 20-5K; General Electric Company, USA) after 2-hour shaking at 200 rpm using a reciprocal shaker (SR-1; TAITEC CORPORATION, Japan). The filtered solutions included water-soluble fraction of cesium. Then, the residue with 2.5 or 5.0 mL of 1M HCl were centrifugally filtered at 18,000 × g for total of 150 min after 2-hour shaking at 200 rpm. The filtered solutions included 1M HCl-soluble fraction of cesium.

Air exchange rate

The air exchange rates in the houses were measured using the CO_2_ decay method. CO_2_ was emitted from gas cylinders in every room of each house, and the indoor air was mixed until the CO_2_ concentration shows similar levels at several points in every room. The indoor CO_2_ concentration was monitored using a CO_2_ monitor (MCH-383SD; Lutron Electronic Co., USA). The outdoor CO_2_ concentration was measured both before and after the indoor CO_2_ measurement. The air exchange rates were obtained by fitting the exponent function to the difference between the monitored CO_2_ concentrations of the indoor and outdoor environments.

Please refer to the previous papers^2,3)^ for further details of methods about cleaning, house dust sampling, aerosol sampling, separation depending on extractability, and air exchange rate.

Evaluation of the differences in the detection efficiency between the certified radioactivity and the samples

The ^137^Cs source is a point source and the shape and size of the samples differ. The smear test paper was round with a diameter of 25 mm. The house dust samples were stored in 2.5-mL cylindrical polypropylene vials with 47 mm in height. Dust samples (<4–20 μm) were sampled on 70-mm polytetrafluoroethylene filters. The filter was folded three-times to make a 1/8 circle for measurement. Solution samples that were separated depending on the extractability were stored in polypropylene cylindrical tubes 94 mm in height. The residue samples remained in the slit (29 mm in width, 5 mm depth, and 15 mm height) of the cylindrical plastic container. The aerosol samples were round with diameters of 25 mm and 37 mm. The discrepancy from the certified radioactivity derived from different shapes between the point source and the samples was measured by placing the point source along the whole circumference of each sample^2,3)^. The largest discrepancy was evaluated from -5.8% to -2.3% among all samples, resulting in the possible underestimation of 2.3% to 5.8% maximum, depending on the type of the sample, since the differences have not been corrected. Note that the differences of efficiency were obtained in extreme cases assuming that the entire radioactivity localize on a specific point in the circumference of each sample. Using a model in which ^137^Cs radioactivities were assumed to be uniformly distributed to the surface of the filter, the efficiency for the filters with diameters of 25 mm and 37 mm was calculated by MCNP5^4)^. The discrepancy in the efficiency for each filter from that of the point source was evaluated as 0% and 0.8%, respectively. The difference in the efficiency was within one standard deviation of 0.32% for the 25 mm phi filter.

Determination of the ^137^Cs radioactivity for the smear and aerosol samples using beta rays

The detection efficiencies corresponding to the beta rays emitted from ^137^Cs are quite higher than those of the HPGe detector for gamma-rays from them. Counts measured with the detector JDC-5300 were from beta-rays emitted from both ^134^Cs and ^137^Cs. For their separation, we have used the measurements with the HPGe detector. By determining the radioactivities of ^137^Cs for smear paper and aerosol filter sources, relationships between radioactivities of ^137^Cs measured with the HPGe detector and net counts measured with the detector JDC-5300 were obtained for both samples with diameters of 25 and 37 mm. Further, the corresponding slope values were evaluated from the relationship as 0.21 cps/Bq (R^2^ = 0.99) and 0.24 cps/Bq (R^2^ = 0.99), respectively, as shown in Supplementary Figs. S5 (a) and (b). The smear test paper and the aerosol filter resemble with a diameter of 25 mm, so that the slope for both was the same. However, it should be noted that there is a difference of 14% in the slopes (the counting efficiency) between filters with diameters of 25 and 37 mm, which might be caused by a small number of measured samples for the filters with a diameter of 37 mm. Hence, the ^137^Cs radioactivities in the 37 mm phi filters could possibly be underestimated by approximately 14% in comparison to those for the 25 mm phi filters. Net counts with JDC-5300 were obtained by subtracting background counts to exclude the effect of natural nuclides. The radioactivity of ^137^Cs, Bq on the aerosol samples was determined by dividing the net count, cps measured with the plastic scintillation counter by the slope value of 0.21 cps/Bq and 0.24 cps/Bq for the samples with 25 mm and 37 mm in diameter, respectively.

Further details for ^137^Cs radioactivity measurement are provided in the previous papers^2,3)^.

Fig. S5 (a)

Fig. S5 (b)

Figs.S5 Relationships between net counts measured with the plastic scintillation counter JDC-5300 and radioactivities of ^137^Cs measured with the Ge detector for filters with diameter of (a) 25 mm and (b) 37 mm, respectively.

Determination of ^137^Cs radioactivity for solution sample extracted from the dust

The radioactivity for solution sample extracted from dust was measured for 100 min with a liquid scintillation counter (LS-6500; Beckman Coulter, Inc., USA) after the addition of a liquid scintillator (4 mL). A relationship between radioactivities measured with the Ge detector and net counts measured with the liquid scintillation counter LS-6500 was obtained and the slope was evaluated from the relationship (Fig. S6). Net counts with LS-6500 were obtained by subtracting background counts to exclude the effect of natural nuclides such as ^40^K.

Please refer to the previous papers^1)^ for further details of methods of determination of ^137^Cs radioactivity for solution sample extracted from dust


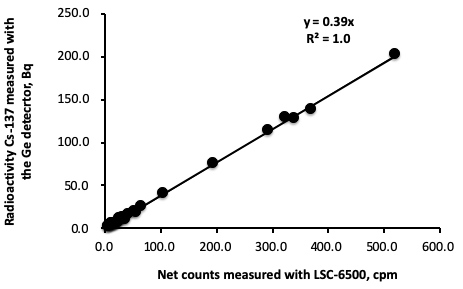


Fig. S6 Relationship between net counts measured with the liquid scintillation counter LS-6500 and ^137^Cs radioactivities measured with the Ge detector.

Determination of indoor contaminants

The total removable surface contamination for beta-emitters, A_sr_ (Bq/cm^2^), was obtained by the following equation^1,5)^,

A_sr_ =(n−n_b_) /(ε_i_ ·ε_s_·F·S) 　　　　 (1)

where n is the gross count rate (sec^−1^), n_b_ the background count rate (sec^−1^), ε_i_ the instrument efficiency, ε_s_ the source efficiency, i.e., the fraction of decays within a sample that result in a particle of radiation leaving the surface of the source, F the removal fraction, and S the surface area covered by the smear (for e.g., 100cm^2^). The product of the instrument and source efficiency, ε_i_·ε_s_ is the counting efficiency^5)^, which was obtained as 0.21 cps/Bq (R^2^ = 0.99) for the smear test paper with a diameter of 25 mm as shown in Supplementary Figs. S5 (a) and (b).

The removal fraction, F, was empirically obtained by the use of repetitive wipes^1,5)^ in real conditions within the houses in evacuation areas as 0.75 ± 0.16. The details corresponding to the determination of the value of F is described in Supplementary Fig. S7.

The detection limit, N_d_ was defined as the analyte count that is required to produce a signal greater than three times the standard deviation of the noise level^6)^.

It is calculated from the following equation,

where N_b_ is the background count rate (min^−1^), and T_s_ and T_b_ are the counting times of the sample and the background (min), respectively, i.e., 10 min for both T_s_ and T_b_.

The lower detection limit for surface contamination was obtained when the smear samples were measured with a plastic scintillator detector JDC-5300. The detection limit for ^137^Cs was evaluated as 0.007 Bq/cm^2^ from equations (1) and (2).

Determination of the value of F

The repetitive wipes were conducted for 67 surfaces within 12 houses in Odaka district, and the towns of Futaba, Okuma, and Tomioka. An area of 100 cm^2^ of the surface of materials of wood, plastic, glass, and metal (34, 14, 12, and 7 surfaces, respectively) were rubbed three times changing a smear test paper^7)^. The ratio, R of n times the removed value of radioactivity to the n-1 times removed value was expressed by the following two equations:

R (n=2) = [(1-F)·AF] / AF = 1 – F (3)

R (n=3) = [A·(1 – F) – [(1-F) ·AF]]·F / [(1-F)·AF]

= [(1 – F - (1-F)·F)·AF] / [(1-F)·AF] = (1 – F)2/(1 – F) = 1 – F (4)

where A is total removal radioactivity.

The relationship between repetitions of the smear and the average ratio of the removed value of radioactivity to the first removed value (n =1) is shown in Fig.S8 with one standard deviation and the equation y = x ^-2^ was fitted to the data.

The value of F, 0.75 ± 0.16 was determined by this relationship and used as F in this study.


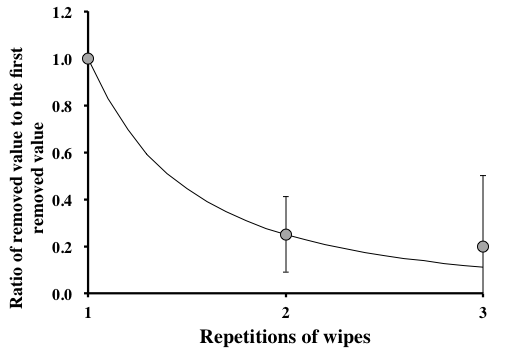


Figs. S7 Relationship between repetitions of the smear and the average ratio of the removed value of radioactivity to the first removed value.

Value of M_soluble_ for each house against distance from the FDNPP

M_soluble_ and M_insoluble_ are the relative ratios of water or 1M HCl soluble fraction to total, and insoluble (the residue) fraction to total, respectively. The sum of M_soluble_ and M_insoluble_ equals one. Note that each value in Fig. S8 corresponds to one at the same distance from the FDNPP in Fig. 5, respectively.


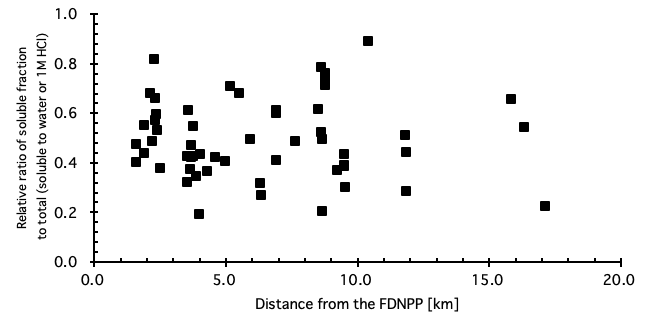


Fig. S8 Value of M_soluble_ for each against distance from the FDNPP.

References

1. JIS Z 4504 Evaluation of surface contamination beta-emitters (maximum beta energy greater than 0.15 MeV) and alpha-emitters (in Japanese) (2008).

Available at: <http://kikakurui.com/z4/Z4504-2008-01.html>

(in Japanese) (Accessed: 31st August 2020)

1. Shinohara, N., Yoshida-Ohuchi, H. Radiocesium contamination in house dust

within evacuation areas close to the Fukushima Daiichi nuclear power plant. Environ.

Int. 114, 107–114. (2018).

Available at:

<https://doi.org/10.1016/j.envint.2018.02.015>

(Accessed: 31^st^ August 2020)

1. Shinohara, N., Yoshida-Ohuchi, H. Radiocesium concentration in indoor air during residential house cleaning in Fukushima Dai-ichi nuclear power plant evacuation areas. J. Environ. Radioact. 205-206, 127-134 (2019).
2. Brown, F. B., Barrett, R. F., Booth, T. E., Bull, J. S., Cox, L. J., Forster, R. A., Goorley, J. T., Mosteller, R. D., Post, S. E., Prael, R. E., Selcow, E. C., Sood, A., and Sweezy, J. ANS Winter Meeting 2002 MCNP Version 5, 1–7, (2002).
3. Frame, P.W., Abelquist, E.W. Use of smears for assessing removable contamination. *Health Phys.* **76**(Supplement 2), S57-S66 (1999).
4. Kaiser, H. Part II Quantitation in Elemental Analysis. *Analytical Chemistry* **424**, 26A–58A (1970).
5. Yoshida-Ohuchi, H., et al., Indoor radiocaesium contamination in residential

houses within evacuation areas after the Fukushima nuclear accident. Sci. Rep. 6,

26412.(2016)

Available at: https://www.nature.com/articles/srep26412

(Accessed: 31st August 2020)
